# Identification of immunogenic HLA-A*02:01 epitopes associated with HCC for immunotherapy development

**DOI:** 10.1097/HC9.0000000000000659

**Published:** 2025-02-26

**Authors:** Anthony Maino, Ekaterina Bourov﻿a-Flin, Thomas Decaens, Saadi Khochbin, Zuzana Macek Jilkova, Sophie Rousseaux, Joel Plumas, Philippe Saas, Laurence Chaperot, Olivier Manches

**Affiliations:** 1 EFS, R&D Department, Grenoble, France; 2 Univ. Grenoble Alpes, INSERM U, CNRS UMR, Institute for Advanced Biosciences, Grenoble, France; 3 Hepato-Gastroenterology and Digestive Oncology Department, CHU Grenoble Alpes, Grenoble, France; 4 PDC*line Pharma SAS, R&D Department, Grenoble, France

**Keywords:** CD8^+^ T-lymphocytes, dendritic cells, epitopes, HCC, T-lymphocyte, tumor-associated antigen

## Abstract

**Background:**

HCC is the most common form of primary liver cancer, and despite recent advances in cancer treatment, it remains associated with poor prognosis and a lack of response to conventional therapies. Immunotherapies have emerged as a promising approach for cancer treatment, especially through the identification of tumor-specific immunogenic epitopes that can trigger a targeted immune response. This study aimed to identify immunogenic epitopes associated with HCC for the development of specific immunotherapies.

**Methods:**

We used high-throughput data screening and bioinformatics tools for antigens and epitope selection. The immunogenicity of the selected epitopes was studied after coculture of peripheral blood mononuclear cells obtained from healthy donors or HCC patients with a plasmacytoid dendritic cell line loaded with the selected peptides. Specific CD8^+^ T cell amplification and functionality were determined by labeling with tetramers and by IFN-γ and CD107a expression (flow cytometry and ELISpot).

**Results:**

We analyzed the transcriptional gene expression landscape of HCC to screen for a set of 16 ectopically expressed genes in a majority of HCC samples. Epitopes predicted to bind to HLA-A*02:01 with high affinity were further validated for their immunogenicity using the previously described plasmacytoid dendritic cell line in ex vivo CD8^+^ activation assays using patient immune cells. Three out of the 30 tested epitopes, namely FLWGPRALV (MAGE-A3), FMNKFIYEI (AFP), and KMFHTLDEL (LRRC46), elicited a strong T-cell response, in activation assays, degranulation assays, and IFN-γ secretion assays.

**Conclusions:**

These results highlight the potential of these peptides to be considered as targets for immunotherapies. The discovery of such immunogenic epitopes should improve immune-based treatments for liver cancer in combination with the current treatment approach.

## Graphical abstract

**Figure:**
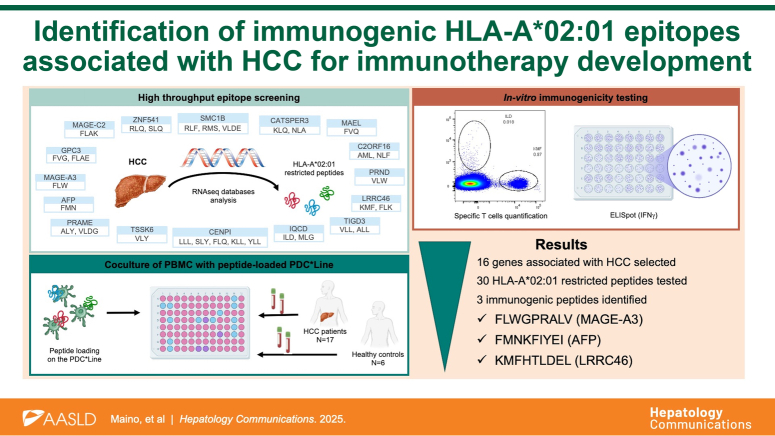

## INTRODUCTION

As the sixth most frequent cancer worldwide and the third cause of cancer-related deaths, HCC is a heavy burden for society. The majority of patients are diagnosed at late stages, limiting the use of surgery or local ablative therapy as a curative treatment.^1^ For these patients with unresectable advanced-stage disease, first-line systemic therapies are now based on immune checkpoint inhibitors combination regimen like atezolizumab/bevacizumab (anti-PD-L1 and anti-VEGF monoclonal antibody) or durvalumab/tremelilumab (anti-PD-L1 and anti-CTLA-4).^2^ These immune checkpoint inhibitors are also considered in early and intermediate stages, but recent clinical trial results demonstrate a modest effect with no clear antitumor immune reaction.^3^ Overall survival remains low, with objective response rates of 20%–30% and a high risk of recurrence,^4^,^5^ emphasizing the need for new treatments.

The liver is characterized by an intrinsic tolerogenic environment allowing limiting responses to food-derived antigens and commensal bacterial products.^6^ In addition, the chronic inflammation associated with fibrosis/cirrhosis conditions in the context of HCC can promote the recruitment of myeloid and regulatory T cells.^7^ Moreover, the tumor microenvironment (TME) is also highly immunosuppressive, with tumor cells secreting IL-10 and TGF-β impairing the activity of both dendritic cells (DCs) and T cells. In HCC, DCs are often immature, with reduced antigen presentation capacities, and poor ability to induce T-cell adaptive immunity.^8^,^9^ Chronic exposure to tumor-associated antigens and expression of PD-L1 by tumor cells also impairs T-cell function, and tumor-infiltrating lymphocytes from HCC samples have been shown to be poorly functional and exhausted, expressing high levels of PD-1, TIM-3, LAG-3, or CTLA-4, and consequently failing to control tumor development.^10^,^11^ Altogether, these pathophysiological mechanisms restrain immunological reactions.

Despite such a tolerogenic environment, specific CD8^+^ T cells directed against HCC antigens have been found in patients and correlated with better survival,^12^,^13^ but their frequency may be too low to efficiently control the disease.^14^ Hence, HCC should be a good candidate for immunotherapies, and stimulating antitumor-specific CD8^+^ T cells could enhance antitumor responses. Besides adaptive cell therapies based on CAR-T cells,^15^ several strategies have been proposed to reinvigorate T-cell responses in HCC patients, including peptide vaccines,^16^,^17^ oncolytic viruses,^18^ and DC vaccines,^19^,^20^,^21^ all providing promising results showing tumor necrosis and development of specific cytotoxic T cells. In 2020, Abdel Ghafar et al^19^ described a therapeutic vaccine based on autologous DCs derived from monocytes and loaded with a protein lysate from an allogeneic hepatic tumor cell line. In this assay, they observed less disease progression for severe patients who received this therapy, compared to patients who received standard care. Nevertheless, because such strategies based on tumor protein lysate can lead to off-target unpredictable immune reactions, some vaccine trials have been carried out to target the few immunogenic antigens that have been described in HCC such as alpha-fetoprotein (AFP) and glypican-3 (GPC-3).^22^,^23^ To this day, one of the main challenges for vaccine development in HCC still relies on identifying reliable antigenic targets that can lead to efficient antitumor responses and tumor rejection.

The identification of relevant antigens involves bioinformatics analyses of HCC databases to select genes that are specifically expressed by tumor cells, coupled with experimental testing of their immunogenicity. Because few immunogenic antigens are known in HCC to this day, we sought to identify new targets based on their restricted expression in the tumor tissue. An interesting class of antigens derives from genes normally restricted to the germline or embryonic stem cells (ESCs) which are re-expressed during oncogenesis due to genetic and epigenetic deregulations. Ectopic gene re-expression massively happens in all types of cancers and may contribute to malignant transformation and immune escape. In addition, the aberrant activation of some of these genes is significantly associated with cancer progression and a poor survival prognosis.^24^ Hence, these genes are interesting targets for immunotherapies as they are usually not or weakly expressed in healthy adult somatic tissues. Based on these findings, a bioinformatics pipeline has been created to identify off-context re-activations and established panels of prognostic biomarkers in several types of cancers.^25^,^26^,^27^,^28^,^29^ Further testing of peptide immunogenicity can be performed using the previously described plasmacytoid dendritic cell line (PDC*line).^30^ Indeed, this cell line is able to generate in vitro robust and functional CD8^+^ T-cell responses against HLA-A*02:01-associated antigenic peptides in the context of various cancer and viral conditions.^31^,^32^,^33^,^34^

The goal of our study was to identify ectopically expressed genes in HCC, and further define immunogenic peptides derived from these genes thanks to the PDC*line technology. With this strategy, we describe identification of new antigenic targets expressed in a sizeable fraction of HCC patients.

## METHODS

### Identification of tissue-predominant genes

Gene selection is aimed at identifying genes having a predominant expression profile in germline and/or ESCs that are not expressed in healthy adult liver tissue. Tissue-specific gene expression profile was determined using RNA-seq data of normal tissues provided by the GTEX portal (https://gtexportal.org) and NCBI Sequence Read Archive (https://www.ncbi.nlm.nih.gov/sra), both consulted in September 2021 and last consulted on September 18, 2024. Reads were aligned using the STAR software.^35^ Aligned reads were counted using the HTSeq framework.^36^ From the read counts, RPKM (Reads Per Kilobase Million) values were calculated and log-transformed by taking log2(1+RPKM). In these datasets of normal tissues, we calculated the average value of gene expression signals in all tissues, and then calculated the Z-score for the tissues in which the expression level was the highest by subtracting the mean from the expression level and then dividing the difference by the SD. Genes were considered to be predominantly expressed in a tissue if the Z-score in this tissue was above 60% of the maximum Z-score. In total, we identified 604 genes predominantly expressed in germline and/or ESCs: 525 of 604 genes were predominant in the testis, 39 genes in the placenta, 39 genes in ESCs, and 1 gene was simultaneously predominant in the placenta and testis (Supplemental Table S1, http://links.lww.com/HC9/B912). For all 604 genes, we calculated the “signal-to-noise” ratio (SNR), corresponding to the ratio between the average expression level in the predominant tissue and the maximum level of expression in other tissues, with higher SNR corresponding to more restricted expression profiles.

### Frequency of aberrant activations in HCC

**FIGURE 1:**
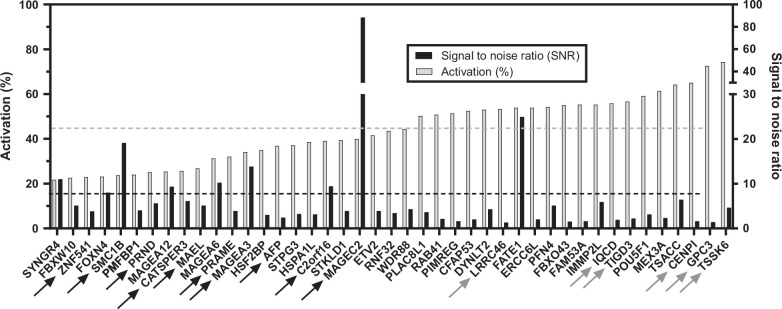
Activation frequencies in HCC (%) and signal-to-noise ratio (SNR) for the 43 preselected genes. Activation frequencies and SNR were calculated from RNA-seq databases. Gray bars represent the percentage of activation frequencies for each preselected gene. Black bars represent the SNR. Genes were ordered to increase the value of activation frequencies. Arrows point to the genes that were selected for rather a high SNR (black arrows) or activation rate (gray arrows).

Next, we examined the number of HCC tissues having an aberrant activation of these genes. To calculate the frequency of aberrant activations in HCC, we used RNA-seq data from the public dataset TCGA-LIHC containing 44 samples of non-tumor liver and 358 HCC samples (https://portal.gdc.cancer.gov/projects/TCGA-LIHC last consulted on September 18, 2024). The normalized FPKM (Fragments Per Kilobase Million) values, downloaded from the TCGA data portal, were log-transformed by taking log2(1+FPKM). For the 604 tissue-specific genes, we established an activation threshold equal to the average value plus 3 SDs of expression levels in non-tumor liver samples. The frequency of aberrant activation corresponds to the percentage of HCC samples for which the expression levels are above the threshold. For a threshold corresponding to activation in at least 20% of samples, 183 genes were selected (Supplemental Table S1, http://links.lww.com/HC9/B912). From this subset, genes with activation in at least 50% of HCC, and genes with <50% expression but with SNR higher than 3 were retained for subsequent immunogenicity tests. AFP was included as it was described before in the literature.^37^ Pseudogenes and noncoding genes were excluded, as well as genes that did not allow the generation of HLA-A*02:01 binding peptides, resulting in a selection of 43 genes (Figure 1). Among them, final selection was performed with combined criteria including the role of the gene in carcinogenesis and genes for which peptides were predicted to have a high affinity for HLA-A*02:01 molecules.

### Bioinformatics prediction of antigenic HLA*02:01 epitopes

After gene screening, the corresponding protein sequences were analyzed to identify potential antigenic peptides. Every possible 9-mer peptides were processed and ranked according to their predicted affinity for HLA-A*02:01 (ANN 4.1-IC50, http://tools.iedb.org/mhci), predicted immunogenicity (http://tools.iedb.org/immunogenicity), proteasome cleavage probability (http://tools.iedb.org/netchop/) and hydrophobicity (Peptide Analyzing Tool, http://thermofisher.com/), consulted on November 15, 2021. To avoid off-target cross-reactivity, we ensured the absence of similarity with peptides derived from other human proteins using the BLASTp software from NCBI (https://blast.ncbi.nlm.nih.gov). Lyophilized peptides were purchased from SmartBiosciences (France), solubilized in dimethyl sulfoxide (Sigma Aldrich) at 4 mM, and stored at −80 °C. Predicted HLA-A2 binding was experimentally validated using an HLA-A2 stabilization assay on T2 cells (CVCL_2211, ATCC). Briefly, the TAP-deficient T2 cell line was incubated in serum-free Roswell Park Memorial Institute (RPMI) 1640 containing Glutamax (Gibco) with the different peptides at 10 µM for 3 hours at 37 °C. Peptide binding was assessed by flow cytometry using anti-HLA-A2 staining (AB_394130, FITC mouse anti-Human HLA-A2, BD Biosciences) and subsequent calculation of the fluorescence index (FI) using the formula (where MFI=median fluorescence intensity): FI=(MFI _(peptide-loaded T2)_−MFI _(unloaded T2)_)/(MFI _(unloaded T2)_). The peptide ELAGIGILTV (MelanA) was used as a positive control for HLA-A2 biding, and the HLA-B*07:02 TPRVTGGGAM peptide (Cytomegalovirus) was used as a negative control.

### Immunogenicity testing

Peripheral blood mononuclear cells (PBMCs) were isolated from whole blood samples using a centrifugation density gradient with lymphocyte separation medium (Eurobio Scientific). Blood samples were obtained from HCC patients (N=17) between December 2022 and June 2023 (biological collection CRB03 #AC-2014-2094, Hospital University). Blood samples from biobanked healthy blood donor volunteers (N=6) collected between February 2021 and March 2023 were used as control (biological collection DC-2019-3803, French Blood Bank). Patients and healthy donors (HDs) gave written informed consent in accordance with the Declaration of Helsinki. HLA-A2 expression was determined on whole blood samples by flow cytometry (AB_394130, BD Biosciences). Cell count and viability were assessed by Acridine Orange/Propidium Iodide staining (Nexcelom Biosciences) with an automated cell counter (Cellometer Auto 2000, Nexcelom Biosciences). Six HCC-derived peptides and 1 positive control peptide were tested per experiment with randomly chosen HCC patients. To test the immunogenicity of the peptides, each peptide was individually loaded on plasmacytoid dendritic cells from the allogeneic PDC*line.^30^ Briefly, this cell line is able to efficiently present antigens associated with HLA-A*02:01 molecules and activate specific CD8^+^ T cells. Peptide-loaded and irradiated PDC*line cells were cocultured with PBMCs in RPMI 1640 containing Glutamax (Gibco) and supplemented with 10% heat-inactivated fetal bovine serum (Gibco), 1.2% nonessential amino acids (Gibco), 1.2% sodium pyruvate (Sigma Aldrich), and 0.2% gentamycin (Gibco). An average of 10.10^6^ PBMCs were cultured for HCC donors and 20.10^6^ for HD. Cocultures were maintained for 21–28 days with weekly restimulation with the peptide-loaded and x-rays-irradiated (60 Gy) PDCs in the presence of IL-2 (200 UI/mL, Proleukin, Clinigen). The ELAGIGILTV peptide from MelanA was used as a positive control. Peptide-specific CD8^+^ T cells were detected by flow cytometry using peptide-specific tetramers combined with FITC-CD3 (AB_395739, BD Biosciences), PerCP-Cy5.5 CD8 (AB_2811219, BD Biosciences) staining. Viability was assessed with Live/Dead staining (L34966, Invitrogen). Tetramers were manufactured from HLA-A*02:01 monomers (Easymer, ImmunAware) incubated with the peptide for 48 hours and further tetramerized with fluorophore-coupled streptavidin 0.2 mg/mL for 1 hour (AB_2868860, BD Biosciences). Data were analyzed with FlowJo software v10.7.2. The cytometry gating strategy successively selected lymphocytes, single cells, live cells, and CD3^+^CD8^+^ cells (Supplemental Figure S1, http://links.lww.com/HC9/B913). A minimal cluster of 20 tetramer-positive cells was defined as the minimum for considering positive staining leading to a detection threshold of 0.05% for an average of 40,000 CD8^+^ T cells present in the positive gate. A positive expansion was defined when at least 2 donors displayed a positive staining (above 0.05%) at day 21.

### Functionality testing

Peptide-specific CD8^+^ T cells expanded with the PDC*line cells were sorted by flow cytometry upon tetramer staining (FACS Aria II). To obtain a monoclonal population, 1 single tetramer-positive cell was dropped per well in RPMI medium containing 10% fetal bovine serum, 300 UI/mL IL-2, 2 µg/mL phytohemagglutinin (R30852801, Thermo Scientific) and feeder cells (100,000 PBMCs, x-rays-irradiated [30 Gy]) from 2 different donors per well and 10,000 ROSI-B-EBV cells (CVCL_A5AU, x-rays-irradiated [60 Gy]) per well). The culture medium was renewed every 2–3 days with a 50% medium change (150 IU/mL IL-2). Cultures were restimulated with a medium containing IL-2, phytohemagglutinin, and feeders every 2 weeks. The functionality of cloned peptide-specific CD8^+^ T cells was assessed by testing IFN-γ secretion by ELISpot according to the manufacturer’s recommendations (Human IFNγ ELISpot Kit, 856.051.002PC, Diaclone). Briefly, 10,000 peptide-specific CD8^+^ T cells were challenged with 20,000 peptide-loaded T2 cells in culture plates during 15–20 hours at 37 °C. Unloaded T2 cells and culture medium were used as a negative control. Stimulation with phorbol-myristate-acetate (P8139, Sigma Aldrich) plus ionomycin (I0634, Sigma Aldrich) was used as a positive control. Spot revelation used a biotinylated detection antibody and Streptavidin-AP substrate with BCIP/NBT. Spots were counted with an automated counter (Bioreader E beta, EazyReader v.20.9) and expressed as a number of spots for 100,000 cells. Using the same conditions, a degranulation assay was performed. Cells were incubated for 6 hours at 37 °C with PE-Cy7 CD107a antibody (AB_10644018, BD Biosciences). Golgi STOP (0.67 µL/mL) (554724, BD Biosciences) was added for the last 4 hours, and tetramer-positive cells were analyzed for CD107a expression by flow cytometry, in combination with CD3, CD8, and Live/Dead staining.

### Statistical analyses

Statistical analyses were performed using GraphPad Prism software (version 9.3.1). Specific CD8^+^ T-cell frequencies of HCC patients and HDs were compared using a non-parametric unpaired Mann–Whitney *U* test. The 2-stage step-up Benjamini, Krieger, and Yekutieli methods were applied to control the false discovery rate.

## RESULTS

### Screening and validation of potential HLA*02:01 epitopes

**FIGURE 2:**
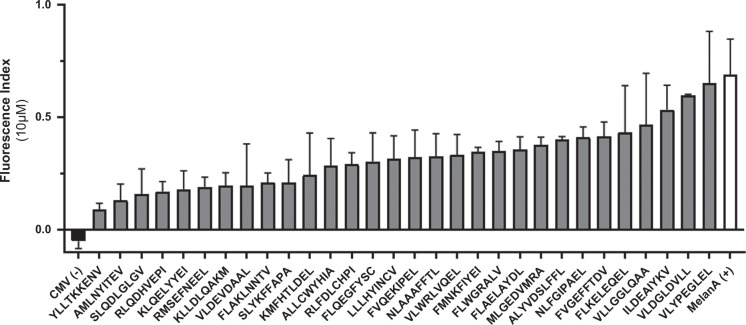
Evaluation of peptide binding to HLA-A2. The fluorescence index was calculated from the median fluorescence intensities of anti-HLA-A2 staining on peptide-loaded (10 µM) and unloaded T2 cells. Cytomegalovirus HLA-B7 restricted peptide was used as negative control (black bar), and MelanA peptide was used as positive control (white bar). Mean with SD of n=4 independent experiments are shown.

In order to identify new and unconventional tumor antigens in HCC, we searched for genes having a differential ectopic expression in HCC and oncogenic potential. We used a comprehensive transcriptomic approach based on RNA-seq databases of normal and tumoral tissues. From genes predominantly expressed in germline/ESCs, we preselected 49 genes having an aberrant expression in at least 20% of HCC samples from databases. The final selection of 15 genes included genes having the highest activation frequencies (>50%) or moderate activation frequencies (20%–50%) but with high tissue restriction (SNR >3) and their role in carcinogenesis along with potential HLA-A2-binding epitopes that could be derived from the protein. The AFP gene that was already described in HCC was also included, resulting in a selection of 16 genes (Figure 1). From these genes, 30 HLA-A*02:01 peptides were selected for immunogenicity testing according to their predicted properties from bioinformatics analyses (Table 1). All peptides had a hydrophobicity lower than 40%, ensuring good solubility, and a predicted IC50 near or inferior to 50 nM suggesting a high affinity for HLA-A2 molecules. Of note, MAGE-C2-derived and PRAME-derived peptides, although presenting lower proteasome cleavage probabilities or predicted immunogenicity were also included based on their previous description in the literature.^38^,^39^ The predicted affinity for HLA-A2 molecules of the panel of peptides was experimentally verified by measuring the HLA-A2 expression level at the surface of the T2 cell line by flow cytometry following incubation with the peptides (10 μM). The FI for selected peptides were all positive, ranging from 0.1 (YLL peptide) to 0.65 (VLY peptide), an intensity similar to the one observed with the positive control MelanA peptide (0.69) showing that the selected peptides were all able to bind HLA-A2 molecules, although at different levels (Figure 2). This result confirmed the binding capacity of the peptides to the HLA-A2 molecule making them suitable for immunogenicity testing.

**TABLE 1:** Characteristics of selected peptides, assessed by bioinformatics tools

Gene	Peptide sequence	IC50^a^ (nmol)	Hydrophobicity^b^	Proteasomal cleavage score^c^	Immunogenicity^d^
CENPI	LLLHYINCV	5.82	32.47	0.68	0.11
	SLYKFFAPA	14.26	33.66	0.34	0.03
	FLQEGFYSC	43.33	27.67	0.20	0.11
	KLLDLQAKM	46.88	26.88	0.71	−0.19
	YLLTKKENV	31.69	23.3	0.93	−0.30
IQCD	ILDEAIYKV	3.58	29.05	0.62	0.14
	MLGEDVMRA	34.91	22.83	0.27	0.05
LRRC46	KMFHTLDEL	13.11	30.57	0.91	0.18
	FLKELEQEL	54.43	31.76	0.86	0.07
TIGD3	VLLGGLQAA	35.93	28.49	0.61	−0.02
	ALLCWYHIA	40.33	38.24	0.30	0.26
TSSK6	VLYPEGLEL	47.17	33.29	0.54	0.17
GPC-3	FVGEFFTDV	34.88	36.09	0.96	0.38
	FLAELAYDL	4.94	38.29	0.49	0.15
MAGE-C2	FLAKLNNTV	6.94	26.59	0.94	−0.20
MAGE-A3	FLWGPRALV	11.96	36.15	0.96	0.17
PRAME	ALYVDSLFFL	6.33	47.91	0.16	−0.01
	VLDGLDVLL	51.09	39.92	0.60	0.08
AFP	FMNKFIYEI	3.2	36.42	0.61	0.08
PRND	VLWRLVQEL	29.9	36.56	0.46	0.11
CatSper3	KLQELYYEI	7.01	31.61	0.95	0.10
	NLAAAFFTL	16.23	36.63	0.02	0.32
C2ORF16	AMLNYITEV	3.18	30	0.14	0.20
	NLFGIPAEL	22.07	35.98	0.04	0.28
SMC1B	RLFDLCHPI	4.84	33.72	0.97	0.02
	RMSEFNEEL	20	24.43	0.66	0.30
	VLDEVDAAL	24.26	26.2	0.53	0.22
MAEL	FVQEKIPEL	34.88	28.64	0.42	0.03
ZNF541	RLQDHVEPI	23.05	21.88	0.07	0.13
	SLQDLGLGV	26.39	28.32	0.08	0.02

^a^ IC50 (http://tools.iedb.org/mhci).

^b^ Hydrophobicity (https://www.thermofisher.com/fr/fr/home/life-science/protein-biology/peptides-proteins/custom-peptide-synthesis-services/peptide-analyzing-tool.html).

^c^ Proteasomal cleavage prediction (http://tools.iedb.org/processing/).

^d^ Immunogenicity score (http://tools.iedb.org/immunogenicity).

### Immunogenicity testing

**FIGURE 3:**
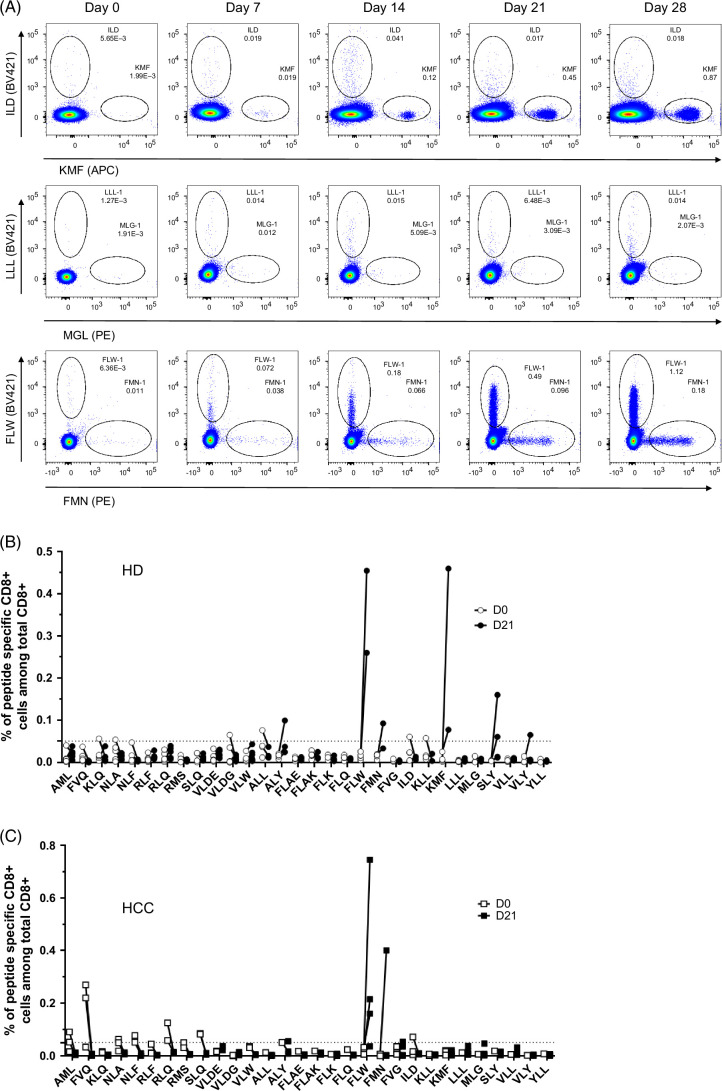
Evaluation of the peptide-loaded PDC*line ability to amplify peptide-specific CD8^+^ T cells in healthy donors and HCC patients. (A) Representative flow cytometry dot plots showing the amplification from day 0 to day 28 of peptide-specific CD8^+^ T cell for 1 healthy donor after coculture with the peptide-loaded PDC*line. Gates are drawn on tetramer^+^CD3^+^CD8^+^ T alive cells. (B, C) Evolution of peptide-specific CD8^+^ T-cell frequencies from D0 (white dots) to D21 (black dots) in healthy donors (panel B, n=4–6) and in HCC patients (panel C, n=3–4). Each dot represents the frequency of peptide-specific CD8^+^ T cells among total CD8^+^ T live cells for each donor or patient. The dashed line represents the limit of the detection threshold of 0.05%. Abbreviation: HD, healthy donor.

To test the immunogenicity of the HCC-derived peptides, defined as their ability to induce peptide-specific CD8^+^ T cells, PBMCs from HD and HCC patients were weekly stimulated in vitro for 21–28 days with the irradiated and peptide-loaded PDC*line. Frequencies of peptide-specific CD8^+^ T cells among total CD8^+^ T cells were assessed weekly by flow cytometry using tetramer staining (Supplemental Figure S1, http://links.lww.com/HC9/B913 and Figure 3A). At baseline, peptide-specific CD8^+^ T cells were barely detectable in both groups. Of note, specific T cells directed against AML (*C2OFR16*), NLF (*C2OFR16*), and FVQ (*MAEL*) peptides were significantly more frequent in HCC donors, but these results should be confirmed in a larger set of patients (Supplemental Figure S2, http://links.lww.com/HC9/B913). In HD, CD8^+^ T cells specific for the 3 peptides FLW (*MAGE-A3*), KMF (*LRRC46*), and SLY (*CENPI*) were efficiently expanded after in vitro stimulation in half of the HD after 21 days of coculture, and peptide-specific CD8^+^ T cells directed against ALY (*PRAME*), FMN (*AFP*), and VLY (*TSSK6*) were expanded for 1 HD (Figure 3B). Of note, the amplification of ALY-specific and VLY-specific CD8^+^ T cells was not considered as positive as the flow cytometry dot plot did not meet the defined quality requirements. In HCC patients, CD8^+^ T cells directed against 2 of these peptides were amplified after 21 days of coculture (FLW and FMN) (Figure 3C). Of note, amplification of FLW-specific CD8^+^ T cells was observed for 3 out of 4 HD. Strikingly, FVQ-specific CD8^+^ T cells that were detected at day 0 in HCC patients could not be amplified with the coculture. The global ability of CD8^+^ T cells from HCC patients to respond to stimulation was verified using the positive control MelanA peptide, and no significant difference was observed between HCC patients and HD in MelanA-specific CD8^+^ T cells expansion using the irradiated and peptide-loaded PDC*line (Supplemental Figure S3, http://links.lww.com/HC9/B913).

### Functionality testing

**FIGURE 4:**
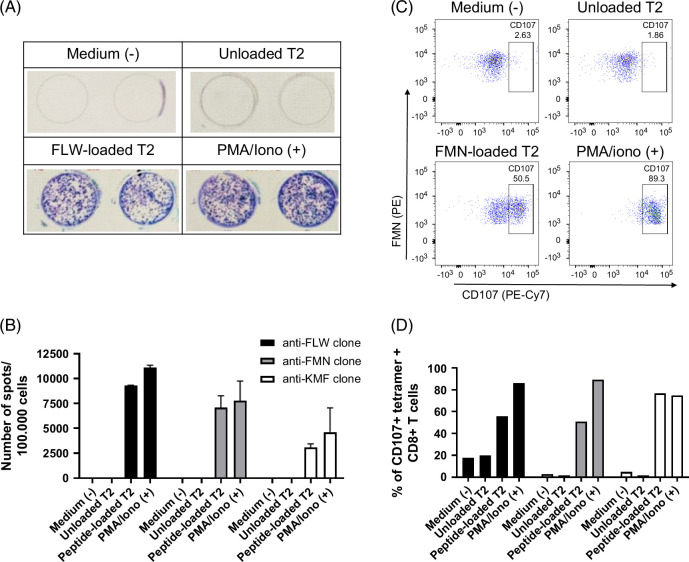
Functionality of T-cell clones. (A) Representative result of IFN-γ ELISpot with CD8^+^ T cells from FLW-specific clone. Upper wells correspond to negative control conditions (medium and unloaded T2). Lower wells correspond to spots detected in the presence of PMA+ionomycin (positive control) or of T2 cells loaded with the FLW peptide. (B) Bar plot showing the number of spots in IFN-γ ELISpot for 100,000 cells upon coculture of T-cell clones directed against FLW (black), FMN (gray), and KMF (white) without or with unloaded or peptide-loaded T2 cells, or with PMA+ionomycin (positive control). The mean and SD of duplicate wells are shown. (C) Representative flow cytometry profiles of CD107a^+^CD8^+^ T cells from FMN-specific clones after stimulation without or with unloaded or FMN peptide-loaded T2 cells or with PMA+ionomycin (positive control). (D) Bar plot showing percentages of CD107a^+^ T cells clones directed against FLW (black), FMN (gray), and KMF (white) after culture without or with unloaded or peptide-loaded T2 cells, or with PMA+ionomycin (positive control) (1 experiment).

To assess the immunological response capacity of the generated T cells, peptide-specific CD8^+^ T cells directed against the FLW (*MAGE-A3*), FMN (*AFP*), and KMF (*LRRC46*) peptides were sorted by flow cytometry, cloned, and then expanded. Two functional assays (ie, IFN-γ ELISpot and CD107a degranulation assays) were used to determine the functionality of expanded CD8^+^ T-cell clones. Challenges of peptide-specific CD8^+^ T cells directed against FLW, FMN, and KMF with their respective peptide-loaded T2 cells (but not with unloaded T2) induced IFN-γ secretion (Figures 4A, B) and CD107a externalization (Figures 4C, D), demonstrating both specificity and functionality of these CD8^+^ T clones directed against these peptides.

## DISCUSSION

In many cancers, the implementation of new antitumoral therapeutic vaccines or T-cell–based strategies depends on the identification of immunogenic and reliable tumor-specific target antigens. Of particular interest in the context of HCC, circulating CD8^+^ T cells directed against common target antigens such as GPC-3, NY-ESO-1, MAGE-A1, and MAGE-A3 have already been detected in HCC patients, but the tolerogenic function of the liver and the immunosuppressive TME have been suggested to interfere with the induction of efficient T-cell responses.^14^ We herein report a method combining bioinformatics and in vitro assays that can be used as a screening platform to identify and define new potential immunogenic epitopes suitable for immunotherapies. In the context of HCC, we tested the immunogenicity of a set of 30 peptides derived from 16 genes having an ectopic expression in HCC. This class of antigens is particularly interesting, as their expression is limited to the tumor besides germinal tissues and/or fetal development stages. Consequently, activation of specific T cells toward these antigens is less likely to induce off-target killing in adult tissues. As physiological exposure to these antigens is low in adults, central and peripheral T-cell tolerance toward these antigens might be less efficient, ensuring unbiased immunological activity.

Our bioinformatics pipeline identified a large number of ectopically expressed genes in a significant fraction of HCC patients, several of which coded for peptides that were demonstrated to bind HLA-A*02:01 molecules with a significant affinity. In vitro, the allogenic peptide-loaded PDC*line cells enabled the amplification of CD8^+^ T cells directed against the 4 peptides FLW (*MAGE-A3*), KMF (*LRRC46*), SLY (*CENPI*), and FMN (*AFP*) peptide. Specific T cells directed against the FMN peptide have also been described in another in vitro study. These cells have been shown to produce IFN-γ and lyse target cells either loaded with or naturally expressing the peptide.^40^ Interestingly, the same authors further connected in vitro findings to in vivo data and described this peptide as immunodominant in HCC in a phase I/II clinical trial that showed that T cells directed against this peptide could expand upon an autologous DC-based vaccination.^37^ Here, we found that 1 HCC patient and 1 HD displayed a significant response against this FMN peptide, hence confirming that this peptide could be an interesting epitope for immunotherapies. This analysis should be expanded to define more precisely the percentage of patients able to mount an anti-FMN-specific T-cell response, estimated to be around one-half of HCC patients.^40^ It has been recently described that the AFP-specific T-cell response tends to increase with the progression of HCC, while specific T-cell response directed toward MAGE-A3 are also detected in early-stage patients.^41^ Interestingly, another study has also shown the immunogenicity of the FLW peptide derived from MAGE-A3 protein, and the amplification of specific CD8^+^ T cells from HDs when stimulated with autologous lymphoblasts.^42^ This protein that is described as a driver of tumor progression in HCC^43^ could constitute another interesting target as RNA-seq analysis in the present study showed its expression in more than 30% of HCC patients.

To the best of our knowledge, the peptides KMF and SLY, respectively derived from the genes *LRRC46* and *CENPI*, have never been described as immunogenic and merit further investigation as novel targets for immunotherapies. We show here that the genes *LRRC46* and *CENPI* are expressed in respectively 53.35% and 65.06% of HCC samples, hence representing new shared HCC-associated antigens. Of note, CENPI has previously been associated with cancer progression.^44^,^45^ Strikingly, those peptides elicited CD8^+^ T-cell responses in HD but not in HCC donors. This was not due to global reduced CD8^+^ T-cell functionality in HCC patients as positive responses to MelanA stimulation were obtained, indicating that they retained their reactivity against peptides. Alternative hypotheses could be that CENPI-specific CD8^+^ T cells have been trapped or inactivated within the tumor, or that circulating precursor frequency among the used PBMC was too low to be detected and to allow amplification. The screening of a larger cohort of HCC patients for their ability to develop CD8^+^ T-cell responses toward these antigens is needed. Besides, the blood sample volume available for this protocol limited the number of PBMCs that were obtained. This limitation might also explain why some peptides such as VLDG and ALY derived from the PRAME protein did not induce positive T-cell responses in our experimental set-up, whereas it has been previously described that CD8^+^ T cells directed against these peptides could be obtained from HDs.^38^ It is likely that beyond the set of proteins investigated here, there exists a large collection of ectopically expressed proteins in HCC with tumor-specific expression that can be targeted by T-cell–mediated immunotherapies.

Notwithstanding the aforementioned limitations, the identification of immunogenic epitopes derived from ectopically expressed proteins in HCC eliciting functional cytotoxic CD8^+^ T cells demonstrates the feasibility of this screening and testing approach. Importantly, these results also demonstrate the functionality of expanded CD8^+^ T cells (cytotoxicity and IFN-γ secretion), reinforcing the interest in targeting the immune response toward these immunogenic peptides in HCC patients. It is also interesting to note that by using the PDC*line cells as antigen-presenting cells, it is possible to overcome the potentially altered functionality of HCC patient’s T cells as described in the context of HBV infection.^32^

Finally, it appears that the implementation and success of such immunotherapies depend on the tumor context and a personalized medicine approach should be considered. Indeed, some predictive factors and biomarkers have been proposed to select patient that should benefit the most of immunotherapies, including TME features, “hot/cold” tumors, stage or etiology of the cancer that could interfere with immune responses.^41^,^46^,^47^,^48^

In summary, we describe here an approach that allows the identification of novel immunogenic epitopes in cancer using in silico analysis and an allogeneic antigen-presenting PDC line. This represents a new tool for the development of future immunotherapies. Applying this method to HCC led to the identification of 4 peptides that merit further evaluation as a combination vaccine targeting this cancer. Although the 4 peptides singled out in this work were predicted to have an extremely high affinity for HLA-A*02:01, in vitro they displayed only median HLA-A2 stabilization capacity compared with other candidate peptides, and no bioinformatics characteristic could straightforwardly predict their immunogenicity. In vitro screening using a highly immunogenic and standardized platform like the PDC*line cells is thus crucial to guide the selection of antigenic peptides in cancer. Although MAGE-A3 is one of the most immunogenic cancer-testis antigens, MAGE-A3 immunotherapeutics (recombinant MAGE-A3 associated with the adjuvant AS15) tested in 2 phase III clinical trials in melanoma and non-small cell carcinoma failed to improve disease-free survival, leading to discontinuation of this treatment development.^49^,^50^ The reasons for the lack of clinical efficacy in these studies could be in part related to the absence of induction of specific T cells. A major challenge for cancer vaccines is to overcome the numerous obstacles to tumor destruction, including tumor heterogeneity, immune escape mechanisms such as loss of HLA-class I molecules or T-cell inactivation by inhibitory immune checkpoints, suppressive cells (regulatory T cells, myeloid-derived suppressor cells), anti-inflammatory cytokines (IL-10, TGF-β) or enzymes (indoleamine 2,3-dioxygenase) present within the TME. The clinical failures of past cancer vaccines raise important questions and suggest that future developments should rely on combination strategies. Indeed, the immunosuppressive action of the TME that reduces the clinical efficacy of vaccines could be reverted by using immune checkpoint blockers, and encouraging early phase clinical trials have tested the efficacy of such combinatorial approaches in cancer patients.^51^ There is certainly also a need to set up clinical trials in patients at early disease stages as immunosuppressive mechanisms increase with disease progression. Improving the formulations, delivery systems, dosages, and routes of administration should also be considered,^52^ and doing so is mandatory to understand the failure of past vaccine trials. Therapeutic vaccines using the PDC*line cells loaded with tumor-associated antigens have already been tested in 2 clinical trials in the context of melanoma (monotherapy, NCT01863108^53^) and lung cancer (combined with Nivolumab, NCT03970746) with very promising results, paving the way for HCC trials using this technology. This approach could also be useful to generate T cells for adoptive therapy or T-cell clones to identify TCR sequences and develop transgenic-TCR T cells. These multiple approaches should reinforce the therapeutic arsenal against HCC and take part in promising combined strategies.

## Supplementary Material

## Data Availability

The data generated in this study are available within the article and the Supplemental Data.
