# Supplementary material for: Identification of immunogenic HLA-A*02:01 epitopes associated with HCC for immunotherapy development
Source: Hepatol Commun. 2025 Feb 26;9(3):e0659. doi: 10.1097/HC9.0000000000000659 (PMC11868434; doi:10.1097/HC9.0000000000000659)
Supplement: Supplementary file 2 [file hc9-9-e0659-s002.docx]

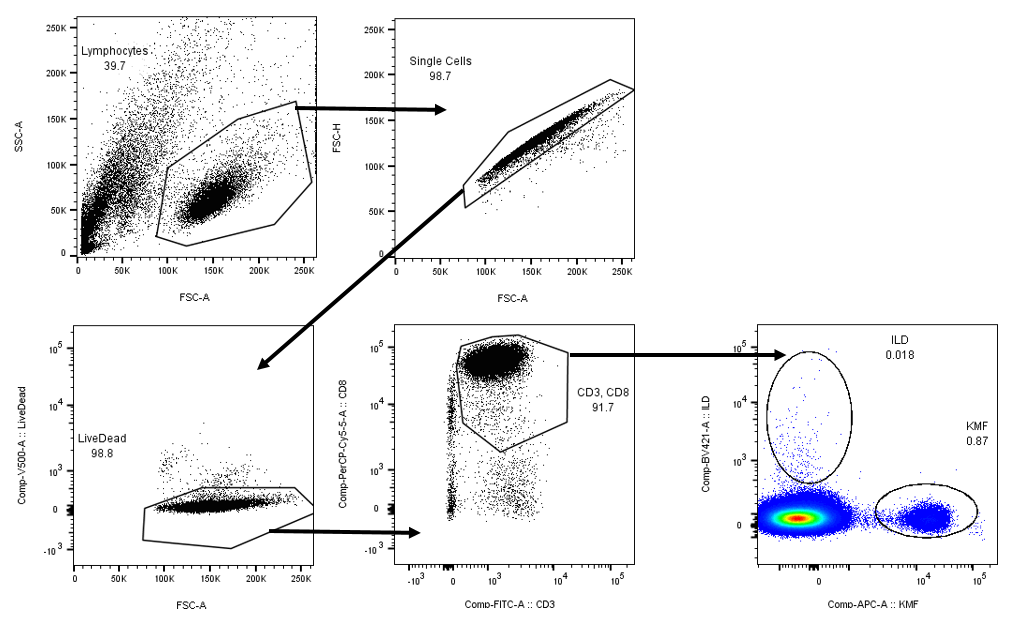


Supplementary figure 1 : Identification of specific T cells.

The flow cytometry strategy consisted on gating on lymphocytes (FSC-A/SSC), single cells (FSC-A/SSC-A), live cells (Live/Dead staining) and CD3^+^CD8^+^ cells. A minimal cluster of 20 tetramer-positive cells were defined as the minimum for considering a positive result.


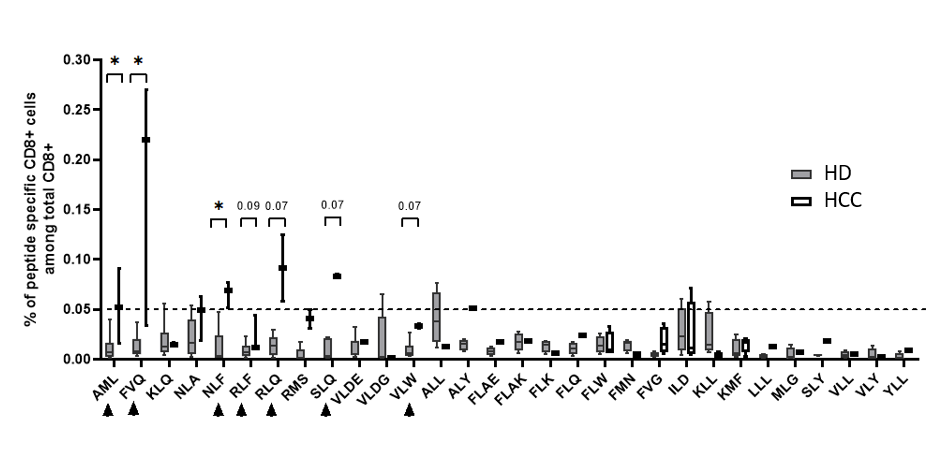


Supplementary figure 2: baseline frequencies of peptide-specific CD8^+^ T cells

Tetramer positive specific CD8+ T cells were quantified ex vivo in PBMC from healthy donors (HD, N=2 to 4) and patients with hepatocellular carcinoma (HCC, N=1 to 4). Statistical analysis was possible and performed for 18 out of 30 peptides in Graph Pad prism, using Mann-Whitney non-parametric test, with two-stage step-up (Benjamini, Krieger, and Yekutieli) method for false-discovery rate control (*: p<0.05 and ns: not significant).


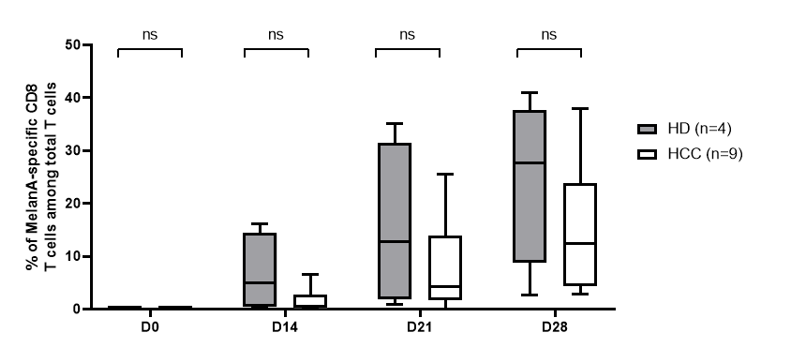


Supplementary figure 3: Amplification of anti-Melan-A specific T cells

Amplifications were compared at day 14, 21 and 28 between cultures performed with PBMC from HD and HCC patients. The global ability of CD8+ T cells to respond to stimulation was similar between HCC patients and HD in MelanA-specific CD8+ T cells upon expansion using the irradiated and peptide-loaded PDC*Line. Comparison using Mann-Whitney test with two-stage step-up (Benjamini, Krieger, and Yekutieli) method for false-discovery rate control (ns: not significant).
